# Draft Genome Sequence of the Novel Enterobacter cloacae Strain amazonensis, a Highly Heavy Metal-Resistant Bacterium from a Contaminated Stream in Amazonas, Brazil

**DOI:** 10.1128/genomeA.00450-18

**Published:** 2018-05-31

**Authors:** Maria Clara Tavares Astolfi, Elen Bethleen de Souza Carvalho, Adriane Menezes de Barros, Marcelo Valente Pinto, Luna Barrôco de Lacerda, Viviane Brito Nogueira, Eraldo Ferreira Lopes, Spartaco Astolfi-Filho

**Affiliations:** a DNA Technology Laboratory, Multidisciplinary Support Center, Federal University of Amazonas, Amazonas, Brazil

## Abstract

Here, we report the draft genome of the Enterobacter cloacae strain amazonensis, a bacterium highly resistant to mercury that was isolated from a metal- and sewage-contaminated stream in Amazonas, Brazil. The exploration of the 5.0-Mb genome revealed 104 genes encoding resistance to toxic compounds and heavy metals, highlighting the potential biotechnological applications of this strain.

## GENOME ANNOUNCEMENT

In the Brazilian Amazon, as an outcome of extensive gold mining, it is estimated that 2000 to 3000 tons of mercury (Hg) was released into the environment (1). Environmental bacteria, evolved from polluted landscapes, encode proteins executing a range of genetic programs to adapt and survive the toxic impact of heavy metals on their metabolism (2, 3). Prospecting for new molecular mechanisms from Amazonian environmental bacteria may provide a powerful informational resource that can lead to novel technologies. To this end, we report here the draft genome of the Enterobacter cloacae strain amazonensis, isolated from a metal- and sewage-contaminated stream named Igarapé do 40 (3.132168S, 60.000696W) in Manaus, Amazonas, Brazil.

Enterobacter cloacae strains are a highly diverse group of Gram-negative *Proteobacteria* belonging to the family *Enterobacteriaceae* (4), which colonize various environments (5–8). For our purposes, we screened urban stream samples for heavy metal tolerance behavior. The amazonensis strain demonstrated a high level of mercury resistance and the ability to tolerate up to 200 mg/liter HgCl_2_ (data not shown), paving the way for potential bioremediation and biotechnological applications of this strain.

We conducted genomic DNA extraction by applying the standard phenol-chloroform method (9). Quality and concentration were verified by Nanodrop and Qubit, respectively. DNA libraries were constructed with qualified DNA through the end-repairing process. We performed sequencing with Illumina QTE HiSeq technology, producing 1 Gb of paired-end reads. Low-quality reads and sequencing adapters were removed using Trimmomatic 0.32 (10). From 1 Gb of data sequencing and 5,629,730 reads, Velvet 1.2.10 (11) and CAP3 (12) were used to perform *de novo* genome assembly, generating a draft genome of 5.0 Mb in 69 contigs. Comparing our draft genome with its reference (13), it presented 90.25% of the size. The estimated *N*_50_ value and G+C content were 327,446 and 53.1%, respectively.

For annotation purposes, we applied Prokka 1.12 (14) software and the RAST web server (15), revealing that the E. cloacae amazonensis genome is composed of 579 subsystems and 4,780 coding sequences, 95 of them for RNAs. Toward heavy-metal metabolism, E. cloacae amazonensis presents 104 genes encoding several molecular tools, such as resistance to arsenic, copper, cobalt, zinc, cadmium, mercury, copper, and chromium. The strain displays distinct genes related to mercury detoxification machinery (*mer*R, *mer*T, *mer*P, *mer*A, *mer*E, *mer*C, and *mer*D) and, interestingly, the genome has two copies of the mercuric reductase gene (*mer*A), which encodes a key enzyme. In a bacterium that is tolerant of a metal-contaminated environment, the duplication of such genetic information may impact the highly Hg-resistant behavior.

### Accession number(s).

This whole-genome shotgun project is available at DDBJ/ENA/GenBank under the accession number PZPP00000000. The version described here is version PZPP01000000.
